# Autologous blood transfusion augments impaired wound healing in diabetic mice by enhancing lncRNA H19 expression via the HIF-1α signaling pathway

**DOI:** 10.1186/s12964-018-0290-6

**Published:** 2018-11-20

**Authors:** Jian-Rong Guo, Lei Yin, Yong-Quan Chen, Xiao-Ju Jin, Xun Zhou, Na-Na Zhu, Xiao-Qian Liu, Han-Wei Wei, Li-Shuang Duan

**Affiliations:** 1 Department of Anesthesiology, Gongli Hospital, the Second Military Medical University, No. 219, Miaopu Road, Pudong New Area, Shanghai, 200135 People’s Republic of China; 2 Ningxia Medical University, Gongli Hospital of Shanghai Pudong New Area Training Base, Shanghai, 200135 People’s Republic of China; 3 Department of Anesthesiology, Yijishan Hospital, 974 the Wannan Medical College, Wuhu, 241001 People’s Republic of China

**Keywords:** Long non-coding RNA H19, EZH2, HIF-1α signaling pathway, Modified autologous blood, Fibroblast activation, Histone methylation, Diabetes mellitus mice, Wound healing

## Abstract

**Background:**

Impaired wound healing frequently occurs in diabetes mellitus (DM) and is implicated in impaired angiogenesis. Long non-coding RNA (lncRNA) H19 has been reported as being reduced in DM and played a critical role in inducing angiogenesis. Thus, we hypothesized that H19 may affect impaired wound healing in streptozotocin (STZ)-induced diabetic mice transfused with autologous blood preserved in standard preservative fluid or modified preservative fluid.

**Methods:**

Fibroblasts in injured skin were isolated and cultured in vitro. After location of H19 in fibroblasts using fluorescence in situ hybridization (FISH), RNA-pull down, RNA immunoprecipitation (RIP), chromatin immunoprecipitation (ChIP), Co immunoprecipitation (COIP) and dual luciferase reporter gene assay were used to verify the binding of H19 to HIF-1α.

**Results:**

The modified preservative fluid preserved autologous blood increased the H19 expression in fibroblasts, and maintained better oxygen-carrying and oxygen release capacities as well as coagulation function. Furthermore, H19 promoted HIF-1α histone H3K4me3 methylation and increased HIF-1α expression by recruiting EZH2. H19 promoted fibroblast activation by activating HIF-1α signaling pathway in fibroblasts and enhanced wound healing in diabetic mice.

**Conclusions:**

Taken together, H19 accelerated fibroblast activation by recruiting EZH2-mediated histone methylation and modulating the HIF-1α signaling pathway, whereby augmenting the process of modified preservative fluid preserved autologous blood enhancing the postoperative wound healing in diabetic mice.

## Background

Diabetic wounds, a severe complication of diabetes mellitus (DM), exert great influence on numerous people globally and it has been found that vascular insufficiency serves as a major reason for impaired wound healing of DM [1]. Impaired wound healing, frequently occurring in DM, is influenced by such factors as impaired glucose metabolism, and neurovascular complications can affect wound healing of DM [2]. Wound healing is considered as a complicated biological process including the following overlapping phases: hemostasis, inflammation, proliferation and remodeling, some of which are associated with impaired wound healing of DM [3]. Impaired wound healing of DM is reported to be involved in reduced production of chemokines, aberrant inflammatory response, as well as impaired angiogenesis [4]. Autologous blood transfusion (ABT) is a collection and reinfusion or transfusion of one’s own blood or blood components before, during or after surgical procedure, whose role in wound healing has been reported [5, 6]. It is known that tissue regeneration with appropriate angiogenesis plays a crucial role in chronic wound healing since blood vessels can provide the sites of tissue regeneration for soluble factors, circulating stem or progenitor cells and nutrients and they can help remove waste products [7]. Recent evidence has shown that long non-coding RNAs (lncRNAs) are important for vascular injury, remodeling and angiogenesis through the regulation of endothelial cells and vascular smooth muscle cells [8].

LncRNAs serve as important factors for various cellular biological processes such as cell proliferation, migration, differentiation, and apoptosis, and are essential for regulating wound healing processes including re-epithelialization, angiogenesis, and scar formation [9]. LncRNA H19, an important imprinted gene locating on human chromosome 11, has been previously reported to promote tenogenic differentiation of tendon-derived stem cells and tendon healing [10]. H19 is also found to enhance proliferation ability of keloid fibroblasts through the regulation of mammalian target of rapamycin (mTOR) and vascular endothelial growth factor [11]. Fibroblasts play crucial roles in wound healing and particularly dermal fibroblasts are significant for skin tissue regeneration [12]. Besides, hypoxia-inducible factor-1α (HIF-1α), a regulator of cell response to inflammation, is found to be expressed in skin wounds, and nitric oxide is a critical factor for the stabilization and function of HIF-1α [13]. HIF-1, a major transcription factor of oxygen homeostasis, in closely related to hypoxic responses which are important for wound healing and remodeling [14]. Notably, HIF-1α transcriptionally activates enhancer of zeste homolog 2 (EZH2) [15], and the histone H3K27 methyltransferase EZH2 facilitates adipogenesis [16]. Moreover, a recent study has reported that autologous platelet-rich plasma, containing fibrin and high concentrations of growth factors, is effective in treating chronic wound healing [17]. Therefore, the present study was conducted in order to identify the effect of lncRNA H19 on postoperative wound healing of in diabetic mice by regulating the HIF-1α signaling pathway.

## Materials and methods

### Ethics statement

The study was conducted under the approval of the Ethics Committee of Gongli Hospital, the Second Military Medical University. All animal experiments in this study conformed to the principles of the management and use of local experimental animals and followed the Guide for the Care and Use of Laboratory Animals published by the National Institutes of Health.

### Model establishment of impaired wound healing in streptozotocin (STZ) induced diabetes mellitus (DM)

A total of 60 male Kunming mice (age: 8 weeks, weight: 26–30 g) were purchased from the Shanghai Laboratory Animal Center of Chinese Academy of Sciences (Shanghai, China) and kept in the clean animal laboratory with constant humidity and temperature. After 1 week of adaptive feeding, the mice were used to establish the diabetes model. The diabetic mouse model was injected by a single intraperitoneal injection of streptozotocin (STZ, Sigma-Aldrich Chemical Company, St Louis MO, USA) with a dose of 70 mg/Kg. The sodium citrate buffer solution (pH = 4.5) was prepared and dissolved into the solution with final concentration of 0.1 mmol/L. Mice were deprived of food more than 8 h a day before modeling, available to water. After injecting STZ, the tail vein blood was drawn from mice. The mice with blood glucose concentration higher than 18 mmol/L were successfully modeled, otherwise the mice were excluded [18].

A model of impaired wound healing was established in mice [19]. After the mice were anesthetized, the hair of the back was shaved and the skin size of 1 cm × 1 cm was cut. The wound size was observed and measured every day, with a total of 16 days [20]. Wound healing was mainly judged by routine hematoxylin and eosin (HE) staining.

After modeling, lentiviral particles of 60 μg/100 μL were immediately intradermally injected into the edge of wound. Specific methods are as follows: a 1 mL sterile syringe was used to extract 200 μL reagent which was injected along the four edges of the wound, with an average of 50 μL on each side. After injection, iodine was used to disinfect gently around the wound skin (not including the surface of wound) and transparent application was used to cover the surface of wound.

The diabetic mice were classified into 6 groups: standard group (diabetic mice were treated with autotransfusion of standard preservative fluid preserved autologous blood); modified group (diabetic mice were treated with autotransfusion of modified preservative fluid preserved autologous blood); sh-H19 group (after injection of lentivirus sh-H19 on the edge of the wound, the diabetic mice were treated with autotransfusion of modified preservative fluid preserved autologous blood); sh-H19 negative control (NC) group (After injection of lentivirus sh-H19 NC on the edge of the wound, the diabetic mice were treated with autotransfusion of modified preservative fluid preserved autologous blood); si-NC group (After injection of lentivirus si-NC on the edge of the wound, the diabetic mice were treated with autotransfusion of modified preservative fluid preserved autologous blood); si-HIF-1α group (After injection of lentivirus si-HIF-1α on the edge of the wound, the diabetic mice were treated with autotransfusion of modified preservative fluid preserved autologous blood). Five mice died in the model group, and 4 mice were unsuccessfully modeled. The rest 51 mice were assigned into 6 groups with 8 mice in each group, and 3 mice were regarded as alternates.

### Preparation of autologous blood preservative fluid

Preservative fluid includes standard preservative fluid and modified preservative fluid. The main components of modified preservative fluid include 2.0 mmol/L adenine, 55.5 mmol/L sugar, 55 mmol/L mannitol, 26 mmol/L sodium chloride, 12 mmol/L disodium hydrogen phosphate, with pH of 6.5. The main components of standard preservative fluid were as follows: 2.2 mmol/L adenine, 110 mmol/L sugar, 70.1 mmol/L sodium chloride, 20 mmol/L disodium hydrogen phosphate, 12 mmol/L citric acid, with pH of 5.8. Compared with the standard preservative fluid, the modified preservative fluid was added with a certain amount of mannitol to stabilize the permeability of the erythrocytes, and reduced the sugar level to avoid the accumulation of the end products of the glycosylation on the erythrocyte membrane, so as to maintain the stability of the erythrocyte membrane and improve the oxygen-carrying function of the diabetic patients.

### Detection of erythrocyte physiological index

A total of 2 mL peripheral venous blood was inserted in the blood vessel containing 2% dipotassium ethylene diamine tetraacetate. German Siemens ADVIA2120 automatic blood analyzer and its matching reagents with ethylenediaminetetraacetic acid (EDTA) as the anticoagulant were used to identify red blood cell (RBC), the hemoglobin (HGB), hematocrit (Hct) and platelet (PLT). Beijing SYSMEX CA530 automatic hemagglutination analyzer and its matching reagents with sodium citrate as the anticoagulant were used to detect prothrombin time (PT), thromboplastin time (APTT), thrombin time (TT), fibrinogen (FIB) and D-dimer (D-D). Danish Radiometer ABL800 automatic blood gas analyzer was used to detect arterial partial pressure of oxygen (PaO_2_) and blood oxygen saturation (SaO_2_).

### Isolation and in vitro perfusion culture of dermal fibroblasts at injury site

Skin at injury site was treated with thermolysin (500 μg·mL^− 1^) to separate dermis and epidermis. Dermal cells were then incubated in the presence of collagenase to release fibroblasts from extracellular matrix. Then the extracted fibroblasts were inoculated in the flask and were grown in the humidified incubator at 37 °C with 5% CO_2_ in the Dulbecco’s modified Eagle’s medium (DMEM, Sigma-Aldrich Chemical Company, St Louis MO, USA) with 10% fetal bovine serum (FBS).

The mature cell perfusion culture system of iBidi company was used to carry out experiments. Fibroblasts were cultured in culture dish. The whole blood (treated with standard or modified preservative fluid) of diabetic mice was treated with perfusion culture at constant speed and then classed into the normal group (fibroblasts treated with standard preservation fluid) and the modified group (fibroblasts treated with modified preservative fluid).

### Cell transfection

The fibroblasts in logarithmic growth phase were treated with trypsin and counted. Then they were seeded in a 6-well plate and diluted with DMEM for transfection. After growing for 24 h, the cells were transfected with Lipofectamine 2000 (Invitrogen Inc., Carlsbad, CA, USA). The mice in each group were blown and mixed, resting for 10–20 min, incubated in an incubator at 37 °C with 5% CO_2_. After transfection, the mice were classified into 8 groups: oe-H19 group, oe-H19 NC group, sh-H19 group, sh-EZH2NC group, sh-EZH2 group, sh-H19 NC group, e-H19 + si-NC group and oe-H19 + si-HIF-1α group.

### Reverse transcription quantitative polymerase chain reaction (RT-qPCR)

The total RNA was extracted by Trizol method. The complementary DNA (cDNA) template was synthesized according to the instructions of High Capacity cDNA Reverse Transcription kit (Applied Biosystems, Foster City, USA). The RT-qPCR experiment was conducted using Power SYBR® Green Master mix (Applied Biosystems, Foster City, USA) and StepOne™ Real-Time PCR System (Applied Biosystems, Foster City, USA). The reaction conditions of lncRNA H19 were as follows: pre-denaturation at 95 °C for 15 min, 40 cycles of denaturation at 95 °C for 15 s, annealing at 56 °C for 20 s, and extension at 72 °C for 30 s, and 3 repeated wells were set for each pair of primers. The conditions for other genes were pre-denaturation at 95 °C for 4 min, 40 cycles of denaturation at 95 °C for 60 s, annealing at 55 °C for 60 s, and extension at 72 °C for 30 s. Three repeated wells were set for each pair of primers. The RT-qPCR primer sequences are depicted in Table 1.

**Table 1:** Primer sequences for RT-qPCR

Gene	Forward sequence	Reverse sequence
H19	5’-GAACAGAAGCATTCTAGGCTGG-3′	5’-TTCTAAGTGAATTACGGTGGGTG-3′
HIF-1α	5’-CGTTCCTTCGATCAGTTGTC-3′	5’-TCAGTGGTGGCAGTGGTAGT-3′
β-actin	5’-TCACCCACACTGTGCCCATCTACGA-3′	5’-CAGCGGAACCGCTCATTGCCAATGG-3′

Note: RT-qPCR, reverse transcription quantitative polymerase chain reaction; HIF-1α, hypoxia inducible factor-1α

### Western blot analysis

Proteins in tissues and cells were extracted, and protein concentration was determined according to the instructions of BCA Kit (Wuhan Boster Biological Technology, Co. Ltd., Wuhan, China). The extracted protein was added to the sample buffer and boiled for 10 min at 95 °C, with 30 μg sample in each well. Then the protein was separated by polyacrylamide gel electrophoresis (PAGE) with the voltage from 80 V to 120 V and wet-transferred onto polyvinylidene fluoride (PVDF) membranes with the voltage of 100 mv for 45–70 min. After being mounted in 5% bovine serum albumin (BSA) for 1 h at room temperature, the membrane was incubated 4 °C overnight with the addition of the following primary antibodies including rabbit polyclonal antibody FAP (1/500, ab53066), alpha 1 chain of type I collagen (Col1A1) (1/1000, ab34710), stromal cell-derived factor (SDF) (1/1000, ab25117), stem cell factor (SCF) (1 μg/mL, ab64677), matrix metalloproteinase (MMP)-1 (1/500, ab137332), MMP-3 (1/500, ab53015), and rabbit monoclonal antibody α-SMA (1/1000, ab32575), vascular endothelial growth factor (VEGF) (1/1000, ab52917), MMP-2 (1/1000, ab92536), HIF-1α (1/1000, ab179483). After being rinsed with TBST for 3 times, 5 min for each time, the membrane was added with the secondary antibody and incubated at room temperature for 1 h, followed by washing for 3 times, 5 min each time. Chemiluminescence reagent was used for developing, and glyceraldehyde-3-phosphate dehydrogenase (GAPDH) served as the internal reference. Bio-rad Gel Dol EZ imager (GEL DOC EZ IMAGER, Bio-rad, California, USA) was used to develop. The gray value of the target protein band was analyzed by Image J software.

### 5-ethynyl-2′-deoxyuridine (EdU) staining

Cells (5 × 10^4^) were seeded into 96-well plates for 48 h, trypsinized and collected. Cells were then incubated for 2 h with the addition of the medium containing EdU, washed with PBS, and then fixed in 4% paraformaldehyde for 30 min. According to the instructions of Edu Kit (Guangzhou RiboBio Co., Ltd., Guangzhou, China), reagents B, C, D, E were added for cell incubation purposes. Next, the cells were washed with PBS, incubated with Hoechst33342 staining solution at room temperature for 30 min, washed again with PBS, and observed under a fluorescence microscope. Statistical analysis was performed using the Image J software.

### Scratch test

The treated cells were inoculated to a 6-well plate. When cell confluence reached about 60%, the pipette gun head was used to scratch in a 6-well plate culture dish. After floating cells were washed with phosphate buffered saline (PBS), the cells were changed into fresh culture fluid, and then cultured for 48 h. After the image were captured, the migration distance of cultured cells was recorded.

### Real-time monitoring for wound healing rate in diabetic mice

The wound healing was recorded on the 0, 8 and 16 days at the established time point by using an in vivo imaging system. Image J was used to quantitatively measure the wound healing area. The percentage of wound healing area to the original wound area represents wound healing rate.

### Immunohistochemical staining

Slices were deparaffinized, hydrated, added with 3% hydrogen peroxide and incubated for 10 min at room temperature, followed by repairing of antigen with microwave method. After being mounted in 5% BSA for 1 h, the slices were incubated in a humidity box at 4 °C overnight with the addition of rabbit monoclonal antibody HSP-47 (fibroblast marker, 1/300, ab109117) [21]. After slices were washed with PBS, biotin-labeled immunoglobulin G (IgG) was added and incubated in a humidity box for 30 min. After that, SP reagent was added and incubated in a humidity box for 60 min. After developing with diaminobenzidine (DAB), the slices were slightly re-stained with hematoxylin, dehydrated, cleared and mounted. In the course of dyeing, PBS or non-immune serum substituted for the first antibody as negative control. Under a microscope (× 400), 10 visual fields were randomly selected for each specimen in the injured area, and the number of positive staining cells in each field was counted.

### Masson staining

After slice deparaffinizing and hydrating, Weigert hematoxylin was used to stain nuclear for 5–10 min, followed by differentiating with hydrochloric acid ethanol for a few seconds. Then the slices were stained with ponceau S acid fuchsin solution for 5–10 min, washed with 2% aqueous solution of glacial acetic acid for a while, differentiated with 1% phospho molybdate aqueous solution for 3–5 min and stained with aniline blue for 5 min. After being washed by 0.2% aqueous solution of glacial acetic acid for a while, the slices were dehydrated, cleared and mounted. Under a microscope (× 400), 10 visual fields were randomly selected for each specimen in the injured area. Image-pro Plus 6 (Media Cybernetics, Inc., Bethesda, MD, USA) image analysis system was used to determine collagen volume fraction (collagen volume fraction = collagen area/total area of wound).

### RNA-pull down

T7 RNA polymerase (Ambion Company, Austin, TX, USA) was transcribed into H19 RNA fragment, treated with RNeasy Plus Mini Kits and DNAse I (Qiagen, Duesseldorf, Germany), and purified using RNeasy Mini Kit. The purified 3’end region of RNA was biotinylated using biotin-labelled RNA complex (Ambion Company, Austin, TX, USA). A total of 1 μg labelled RNA was heated to 95 °C in RNA structure buffer (10 mmol/ L Tris pH 7, 0.1 mol/L KCl, and 10 mmol/L MgCl_2_). After 2 min, RNA was incubated on ice for 3 min and allowed to stand at room temperature for 30 min to form appropriate RNA secondary structure. After that, 3 μg THP-1 macrophage derived foam cells were added into cell lysate (Sigma-Aldrich, St. Louis, MO, USA) and lysed at 4 °C for 1 h. The lysate was centrifuged at 12000×g at 4 °C for 10 min and transferred into a RNAse-free centrifugal tube after the collection of supernatants. Subsequently, 400 ng biotinylated RNA were added into 500 μL RNA immunoprecipitation (RIP) buffer and incubated with cell lysate at room temperature for 1 h. Afterwards, the streptavidin magnetic beads were added into each binding reaction and incubated at room temperature for 1 h. Eventually, the magnetic beads were washed with RIP buffer 5 times and incubated with the addition of 5 × loading buffer at 95 °C for 5 min to detect the eluted proteins by western blot analysis using the following rabbit anti-EZH2 (1: 2000, ab186006), H3K4me3 (1: 1000, ab8580), H3K27me3 (1: 1000, ab192985), H3K9me3 (1: 1000, ab8898), and H3K36me3 (1: 1000, ab9050). These antibodies all from Abcam Inc., (Cambridge, MA, UK).

### Rip

The procedures were conducted in accordance with the instructions of the Magna RIP RNA-Binding Protein Immunoprecipitation reagent kit (Merck Millipore, Billerica, MA, USA). The cells were collected using cell scrape, washed twice with pre-cooled PBS, and lysed with 100 μL lysis buffer containing proteinase inhibitors and ribonuclease inhibitors on ice for 30 min. After centrifugation at 120000 g at 4 °C for 30 min, the supernatant was transferred into a centrifuge tube. IgG was taken as the negative control. Next, 1 μg corresponding antibodies: rabbit anti-EZH2 (1: 500, ab186006, Abcam Inc., Cambridge, MA, UK) and 10 ~ 50 μL protein A/G-beads were added to the remaining supernatant, and incubated overnight at 4 °C. After immunoprecipitation, centrifugation with 3000 g at 4 °C for 5 min was conducted, the supernatant discarded. The protein A/G-beads were then washed with 1 mL lysis buffer, and deposited 3 ~ 4 times. After each washing, the sample was centrifuged with 1000 g at 4 °C for 1 min. Finally, 2 × sodium dodecyl sulfate (SDS) buffer (15 μL) was added, and then heated in the boiled water for 10 min. Next, the relative RNA was obtained by isolation and purification from the precipitation. The binding effect of lncRNA H19 bound to EZH2 and H3K4me3 was identified using the specific primers of lncRNA H19 and RT-qPCR. The primers of H19: forward: 5’-ACTGCACTACCTGACTCAGGAAT-3′, reverse: 5’-AAGAGACAGAAGGATGAAAAAGA-3′.

### Co immunoprecipitation (COIP) assay

The cells were washed with precooled PBS 5 times and the PBS was thoroughly sucked at the last time of washing. According to the area of the culture dish, cells were added with precooled Co-IP Lysis Buffer (20 mM Tris [pH = 8.0], 150 mM NaCl, 0.5% NP-40, 10% Glycerol, 2 mM EDTA) (freshly added with Cocktail protease inhibitor and phosphatase inhibitor) and placed on ice for 5 min. The cells scraped by cell scraping were transferred into a precooled eppendorf (EP) tube, lysed at 4 °C for 30 min, centrifuged at 12000 g for 20 min, and transferred into a new EP tube. After that, the Protein G agarose beads (#11719233001, Roche, Mannheim, Germany) were rinsed with precooled PBS, activated three times at 2000 g for 1 min at 4 °C, during which, the pipette tip should be cut off so as not to damage the beads. Every 1 mL total protein was added with 100 μL Protein G agarose beads (50%) and oscillated at 4 °C for 10 min to remove the background. And then, the proteins were centrifuged at 12000 g at 4 °C for 20 min and the supernatants were transferred into a new EP tube, followed by the detection of the protein concentration. With the addition of primary rabbit anti-EZH2 (1: 500, ab186006, Abcam, Cambridge, MA, UK), the antigen antibody mixture was slowly oscillated at 4 °C for 4–6 h, and oscillated at 4 °C overnight with the addition of 100 μL activated Protein G agarose beads (50%). After centrifugation at 2000 g for 1 min at 4 °C, the antigen antibody and the Protein G agarose beads were collected. After the removal of the supernatants, the proteins were washed with precooled Co-IP Lysis Buffer three times (800 μL buffer/time), centrifuged at 2000 g for 1 min at 4 °C, mixed with 60 μL 1.5 × Co-IP loading buffer, and boiled at 100 °C for 10 min. The subsequent procedures were the same with those of western blot analysis. The H3K4me3 content was detected using western blot analysis with H3K4me3 (1: 1000, Abcam, ab8580, UK) as the antibody.

### Fluorescence in situ hybridization (FISH)

FISH was employed to determine the localization of CDKN2B-AS1 in fibroblasts according to the instructions of the RiboTM lncRNA FISH Probe Mix (Red) (Guangzhou RiboBio Co., Ltd., Guangzhou, Guangdong China). The CDKN2B-AS1 probe was prepared based on the CDKN2B-AS1 and the specific procedures were as follows: the coverslip was put in a 6-well plate and the THP-1 macrophage derived foam cells were inoculated into the plate. After 1 day of cell culture, when cell confluence reached about 80%, the coverslip was taken out and the cells were washed with PBS three times and fixed with 1 mL 4% polyformaldehyde at room temperature. After treatment with protease K (2 μg/mL), glycine, and acetylation reagent, the cells were incubated with 250 μL prehybridization solution at 42 °C for 1 h. After removal of prehybridization solution, cells were added with 250 μL hybridization solution containing probe (300 ng/mL) and hybridized at 42 °C overnight. After the cells were washed with Phosphate-Buffered Saline/Tween (PBST) three times, the nucleus was stained using 4′,6-Diamidino-2-Phenylindole (DAPI) diluted by PBST at the ratio of 1: 800. Subsequently, the cells were added into the 24-well culture plate, stained for 5 min, and washed with PBST three times, 3 min each. After the coverslip was mounted using the anti-fluorescence quenching agent, the images of cells were captured under 5 different visual fields using the fluorescence microscope (Olympus Optical Co., Ltd., Tokyo, Japan) [22].

### Chromatin immunoprecipitation (CHIP)

The binding of the HIF-1α gene promoter to H3K4me3 was verified in accordance with the instructions of the ChIP kit (Merck Millipore, Billerica, MA, USA). We used 1% formalin to fix cells for 10 min to facilitate DNA and protein cross-linking. Next, the random fragmentation of DNA to 200–800 bp was conducted by ultrasonic, and DNA was immunoprecipitated with the target protein specific antibodies: H3K4me3 (ab8580, rat antibody, Abcam Inc., Cambridge, MA, USA) and IgG in the control group. Finally, 100 μl H_2_O was used to purify and elute ChIP DNA and 2.5 μL ChIP DNA was obtained for RT-qPCR detection. The binding of the HIF-1α gene promoter and H3K4me3 was detected by HIF-1α primers.

### Dual luciferase reporter gene assay

A base complementary pairing site at the promoter region of the H19 to HIF-1α gene was detected by the Basic Local Alignment Search Tool (BLAST) and verified with dual luciferase reporter gene assay. Fibroblasts were co-transfected with plasmids DNA (HIF-1α-wild type (WT) or HIF-1α-mutant (MUT), H19 (50 nM) or H19 negative control (H19 NC)). Following a 24-h period of transfection, the experiment was carried out in accordance with the multifunctional enzyme labeling apparatus based on the instructions of the dual luciferase reporter gene assay kit (BioTek, Winooski, VT, USA). Firefly luciferase and renilla luciferase were used to assess the relative luciferase activity. The experiment was repeated 3 times.

### Statistical analysis

All data analysis was conducted using SPSS 19.0 software (IBM Corp. Armonk, NY, USA). The measurement data were presented as the mean ± standard deviation. Comparisons between two groups were analyzed by unpaired *t*-test (independent-sample *t*-test), when data were normally distributed with homogeneity of variances. If the data were not normally distributed with heterogeneity of variance, Welch’s *t*-test was performed. Repeated measurement analysis of variance was used to compare values at different time points. Statistical significance was defined as *p* < 0.05.

## Results

### Transfusion of modified preservative fluid preserved autologous blood promotes H19 expression in fibroblasts in diabetic mice

The blood of diabetic mice was preserved in modified preservative fluid or standard preservative fluid for different days, and the indexes related to oxygen-carrying function of red blood cells were detected. The results were as follows: compared with preservation with the modified preservative fluid for 7 days, the blood of diabetic mice preserved with modified preservative fluid for 21 days showed significantly reduced RBC, HB, Hct, PaO_2_ and PLT, while no significant difference in SaO_2_. After preservation for 7 days and 21 days by standard preservative fluid, RBC, HB, Hct, PaO_2_ and PLT were significantly decreased while SaO_2_ did not change significantly in comparison to preservation for 7 days by the modified preservative fluid (Table 2). The above results showed that compared with whole blood preserved by modified preservative fluid for a long period of time (21 days) and whole blood preserved by standard preservative fluid, the whole blood preserved by acid additive for a short period of time (7 days) could better maintain the oxygen-carrying capacity and oxygen release of the RBCs in vitro.

**Table 2:** The influence of preservation by modified or standard preservative fluid for different days on oxygen-carrying function of the blood in diabetic mice

Index	Modified preservative fluid		Standard preservative fluid	
	7 d	21 d	7 d	21 d
RBC × 10^12^/L	4.75 ± 0.65	2.59 ± 0.53^a^	3.15 ± 0.60^a^	1.32 ± 0.42^a^
HB g/L	129.63 ± 19.41	87.16 ± 19.47^a^	103.32 ± 18.51^a^	67.4 ± 12.12^a^
Hct vol/%	45.21 ± 1.14	26.07 ± 1.03^a^	38 ± 1.53^a^	21 ± 1.49^a^
PaO_2_/Kpa	15.43 ± 2.1	9.45 ± 2.46^a^	13.92 ± 3.19^a^	7.36 ± 1.85^a^
SaO_2_/%	97.62 ± 0.69	96.34 ± 0.53	96.57 ± 0.77	95.29 ± 0.79
PLT × 10^9/L	224.08 ± 22.56	114.48 ± 21.9^a^	147.43 ± 13.86^a^	74.45 ± 19.12^a^

Note: *DM* diabetes mellitus, *d* days, *RBC* red blood cell, *HB* hemoglobin, *Hct* hematocrit, *PaO*_*2*_ partial pressure of oxygen, *SaO*_*2*_ blood oxygen saturation, *PLT* platelet; ^a^, VS. preservation of the modified preservative fluid or the standard preservative fluid for 7 days, *P* < 0.05

The influence of diabetic mouse blood preserved by modified preservative fluid and standard preservative fluid for different days on the coagulation index was detected. The results showed that, compared with preservation by modified preservative fluid for 7 days, diabetic mice blood preserved by modified preservative fluid for 21 days had significantly reduced FIB, significantly increased PT and D-D, and no significant change in APTT and TT. Compared with preservation by modified preservative fluid for 7 days, FIB significantly decreased, and PT and D-D increased significantly, while APTT and TT did not change significantly after preservation for 7 days and 21 days by standard preservative fluid (Table 3). The above results showed that compared with whole blood preserved by modified preservative fluid for a long period of time (21 days) and whole blood preserved by standard preservative fluid, the whole blood preserved by modified preservative fluid for a short period of time (7 days) could better maintain coagulation ability.

**Table 3:** The influence of preservation by modified or standard preservative fluid for different days on coagulation function of the blood in diabetic mice

Index	Modified preservative fluid		Standard preservative fluid	
	7 d	21 d	7 d	21 d
PT/s	9.62 ± 1.39	14.29 ± 2.39^*^	13.56 ± 1.17^*^	15.4 ± 2.01^*^
APTT/s	30.29 ± 9.88	33.2 ± 12.33	31.71 ± 11.95	32.89 ± 15.21
TT/s	13.20 ± 2.36	14.28 ± 3.16	15.85 ± 2.79	14.34 ± 2.64
FIB g/L	3.41 ± 0.95	1.7 ± 0.36^*^	2.32 ± 0.61^*^	1.02 ± 0.53^*^
D-D mg/L	0.43 ± 0.13	0.85 ± 0.19^*^	0.54 ± 0.23^*^	1.04 ± 0.15^*^

Note: *DM* diabetes mellitus, *d* days, *PT* prothrombin time, *APTT* activated partial thromboplastin time, *TT* thrombin time, *FIB* fibrinogen, *D-D* D-dimer; ^*^, VS. preservation of the modified preservative fluid for 7 days, *P* < 0.05

**Fig. 1:**
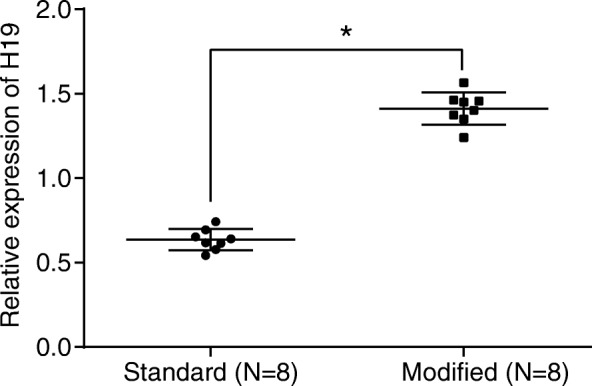
H19 expression was increased in diabetic mouse when the whole blood was preserved by modified preservative fluid. *, *p* < 0.05, compared with the standard preservative fluid; standard, whole blood of diabetic mice preserved in standard preservative fluid; modified, whole blood of diabetic mice preserved in modified preservative fluid; The results of the RT-qPCR were measurement data, which were presented as the mean ± standard deviation. Comparisons between two groups were analyzed by *t*-test. The experiment was repeated 3 times; RT-qPCR, reverse transcription quantitative polymerase chain reaction

The lncRNA H19, which was first identified from gene screening in 1984 [23], plays an important role in triggering angiogenesis [24]. Interestingly, the expression of lncRNA H19 in DM was significantly reduced [25, 26]. Furthermore, previous study pointed out that lncRNA H19 was indispensable for vascular regeneration [1]. In order to detect the effect of whole blood preserved by modified preservative fluid on H19 expression in fibroblasts of diabetic mice, the blood of diabetic mice was preserved by modified preservative fluid or standard preservative fluid respectively for 7 days, followed by transfusion of skin-injured diabetic mice and extracting the dermal fibroblasts from the wound. The changes of H19 expression were detected by RT-qPCR. The results showed that compared with the whole blood preserved in the standard preservative fluid, diabetic mice with transfusion of whole blood preserved by modified preservative fluid had significantly increased H19 expression in fibroblasts (Fig. 1).

### H19 promotes the activation of fibroblasts in diabetic mice

**Fig. 2:**
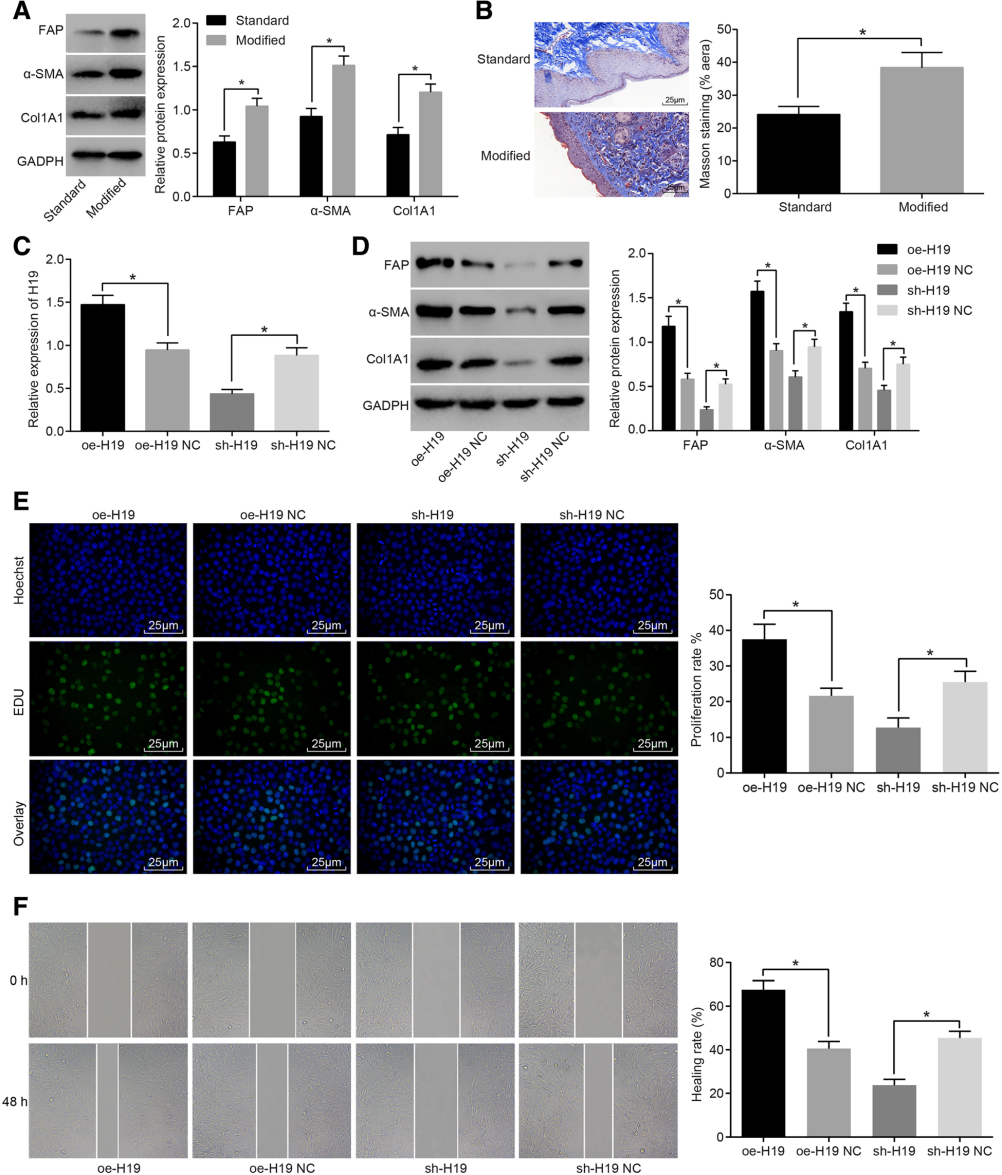
Modified preservative fluid preserved autologous blood enhanced the activation of fibroblasts in diabetic mice through up-regulating H19 expression. **a** western blot analysis showing that modified preservative fluid preserved autologous blood increased the expression of fibroblast activation marker proteins (FAP, Col1A1, α-actin and α-SMA); **b** Masson staining (× 400) showing that modified preservative fluid preserved autologous blood increased collagen content of skin tissues; scale bar = 25 μm; *N* = 8; **c** RT-qPCR showing that expression of H19 in fibroblasts was significantly increased/decreased after transfection of H19 overexpression/siRNA H19 plasmids; **d** western blot analysis showing that expression of fibroblast activation related proteins (FAP, Col1A1 and α-SMA) was significantly increased/decreased in response to H19 overexpression/siRNA H19 transfection; **e** EDU staining (× 400) showing that the proliferation ability of fibroblasts was significantly increased/decreased in response to H19 overexpression/siRNA H19 transfection; scale bar = 25 μm; **f** Scratch test showing that the migration ability of fibroblasts was significantly increased/decreased in response to H19 overexpression/siRNA H19 transfection; *, *p* < 0.05; The results were measurement data, which were presented as the mean ± standard deviation. Comparisons between two groups were analyzed by *t*-test. The experiment was repeated 3 times; standard, standard preservative fluid preserved autologous blood; modified, modified preservative fluid preserved autologous blood; RT-qPCR, reverse transcription quantitative polymerase chain reaction; DM, diabetes mellitus; NC, negative control; α-SMA, α-smooth muscle actin; EDU, 5-ethynyl-2′-deoxyuridine

In order to detect the role of modified preservative fluid preserved autologous blood in the activation of diabetic fibroblasts, diabetic mice were treated with modified preservative fluid or standard preservative fluid for 7 days. The dermal fibroblasts were extracted from the wound, and the expression of fibroblast activation related proteins was detected by western blot analysis. The results showed that compared with the standard preservative fluid, the autologous blood stored in the modified preservative fluid could significantly increase the expression of fibroblast activation marker proteins FAP, Col1A1, α-actin and α-SMA (Fig. 2a). The collagen content of the skin tissue of diabetic mice was detected by Masson staining. The results showed that modified preservative fluid preserved autologous blood could significantly increase collagen content of skin tissues compared with the standard preservative fluid (Fig. 2b). These results indicated that modified preservative fluid preserved autologous blood could promote the activation of fibroblasts in DM.

We isolated the fibroblasts of the injured skin, and conducted in vitro perfusion culture under high glucose conditions after silencing and overexpressing H19 expression respectively, in order to validate the regulation of modified preservative fluid preserved autologous blood on the activation of fibroblasts in DM through up-regulating H19 expression. The expression of H19 in the fibroblasts after transfection was detected by RT-qPCR. The results showed that H19 expression of fibroblasts in the oe-H19 group increased significantly compared with the oe-H19 NC group, and the H19 expression in the sh-H19 group was significantly lower than that in the sh-H19 NC group (Fig. 2c). Western blot analysis was used to detect the expression of activation related protein in the fibroblasts after transfection. The results showed that the expression of FAP, Col1A1 and α-SMA in the oe-H19 group was significantly higher than that of the oe-H19 NC group. Compared with the sh-H19 NC group, the expression of FAP, Col1A1 and α-SMA in the sh-H19 group was significantly decreased (Fig. 2d). EDU staining was employed to assess the proliferation ability of the transfected fibroblasts. The results showed that the proliferation ability of fibroblasts in the oe-H19 group increased significantly compared with the oe-H19 NC group, and the proliferation ability of fibroblasts in the sh-H19 group was significantly lower than that in the sh-H19 NC group (Fig. 2e). At the same time, we detected the migration ability of fibroblasts by scratch test. The results showed that the migration ability of fibroblasts in the oe-H19 group increased significantly compared with the oe-H19 NC group, and the migration ability of fibroblasts in the sh-H19 group was significantly lower than that of the sh-H19 NC group (Fig. 2f). The above results showed that the diabetic mice with skin injury were treated with transfusion of modified preservative fluid preserved autologous blood and up regulation of H19 expression could significantly promote fibroblast activation.

### Modified preservative fluid preserved autologous blood promotes wound healing in diabetic mice through up-regulating H19 expression

**Fig. 3:**
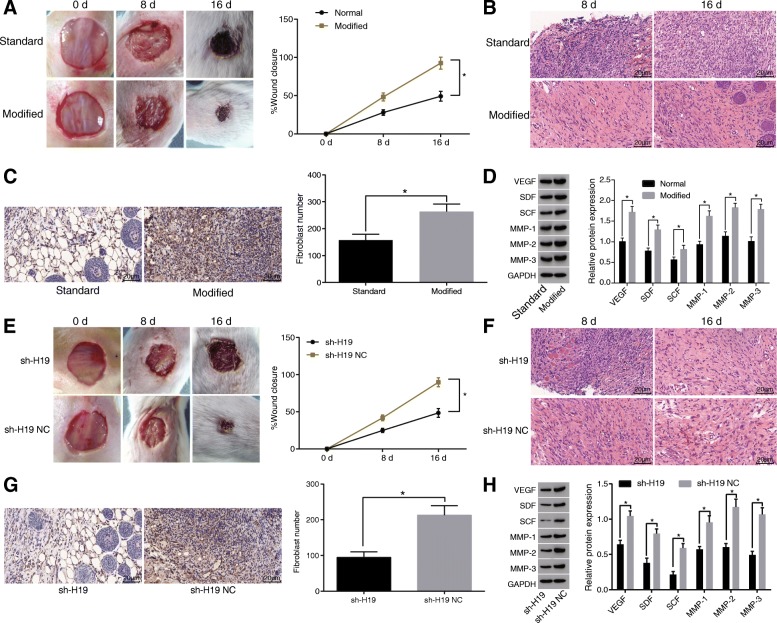
Modified preservative fluid preserved autologous blood promotes wound healing in diabetic mice through up-regulating H19 expression. **a**, the modified preservative fluid preserved autologous blood could significantly increase the speed of wound healing in diabetic mice; N = 8; **b** HE staining (× 400) was used to observe histopathological changes during wound healing at different time points in response to standard and modified preservative fluid preserved autologous blood; scale bar = 25 μm; **c** immunohistochemical staining (× 400) showing that modified preservative fluid preserved autologous blood in diabetic mice could significantly increase the number of fibroblasts; scale bar = 25 μm; N = 8; **d** western blot analysis was used to detect the expression of wound healing related proteins, showing that modified preservative fluid preserved autologous blood significantly elevated the expression of VEGF, SDF, SCF, MMP-1, MMP-2 and MMP-3; **e** speed of wound healing was inhibited after injection of sh-H19 lentivirus; *N* = 8; **f** HE staining (× 400) was used to observe histopathological changes during wound healing at different time points in response to injection of sh-H19 lentivirus; scale bar = 25 μm; **g** immunohistochemical staining (× 400) showing that number of fibroblasts was decreased after injection of sh-H19 lentivirus; scale bar = 25 μm; *N* = 8; **h** western blot analysis showing that the expression of VEGF, SDF, SCF, MMP-1, MMP-2 and MMP-3 was reduced in response to injection of sh-H19 lentivirus; *, *p* < 0.05; The above results were measurement data, which were presented as the mean ± standard deviation. Comparisons between two groups were analyzed by *t*-test. The speed of wound healing was analyzed by two-way analysis of variance. The experiment was repeated 3 times; standard, standard preservative fluid preserved autologous blood; modified, modified preservative fluid preserved autologous blood; DM, diabetes mellitus; NC, negative control; VEGF, vascular endothelial growth factor; SDF, stromal cell-derived factor; SCF, stem cell factor; MMP, matrix metalloproteinase; GAPDH, glyceraldehyde-3-phosphate dehydrogenase; HE, hematoxylin-eosin

In order to detect the effect of modified preservative fluid preserved autologous blood on wound healing in diabetic mice, we examined the speed of wound healing in diabetic mice. The results showed that compared with the standard preservative fluid, the modified preservative fluid preserved autologous blood could significantly increase the speed of wound healing (Fig. 3a). At the same time, HE staining was used to observe the skin tissues of the wound on the 8 day and 16 day (Fig. 3b). The results showed that compared with the standard preservative fluid, a large number of inflammatory cells were found in the wound 8 days after transfusion of the modified preservative fluid preserved autologous blood in diabetic mice; besides, plenty of fibroblast-like cells began to appear. On the 4–8 days after injury, a large number of new blood vessels were found in the wound, combined with fibroblast-like cells and inflammatory cells forming granulation tissue. On the 8–16 days after injury, the new blood vessels in the injured area decreased significantly, the interstitium increased and the granulation tissue changed to the scar tissue. Immunohistochemical staining results of HSP (fibroblast marker) showed that the modified preservative fluid preserved ABT in diabetic mice could significantly increase the number of fibroblasts compared with the standard preservative fluid (Fig. 3c). Western blot analysis detected the expression of wound healing related proteins. The results showed that the modified preservative fluid preserved ABT in diabetic mice could significantly promote the expression of VEGF, SDF, SCF, MMP-1, MMP-2 and MMP-3 compared with the standard preservative fluid (Fig. 3d). The above results showed that modified preservative fluid preserved autologous blood could promote angiogenesis and the migration of fibroblasts, and increase the secretion of extracellular matrix, thereby promoting wound healing.

In order to detect modified preservative fluid preserved autologous blood promoting wound healing in diabetic mice through up-regulating H19 expression, sh-H19 lentivirus was injected into the injured skin of diabetic mice and the speed of wound healing was detected. The results showed that the wound healing speed of the sh-H19 group was significantly slower than that of the sh-H19 NC group (Fig. 3e). At the same time, HE staining was used to observe the histopathological changes at each time point after the wound (Fig. 3f). The results showed that compared with the sh-H19 NC group, in the sh-H19 group the inflammatory cell infiltration was found, which reached the peak on the 8th day after injury, and a large number of inflammatory cells were still found on the 16th day after injury; granulation tissue was formed 8–16 days after injury, as well as fibroblast-like cells and new blood vessels. The results of immunohistochemical staining of HSP (fibroblast markers) showed that the number of fibroblasts in the sh-H19 group was significantly lower than that in the sh-H19 NC group (Fig. 3g). The expression of wound healing related protein was detected by Western blot analysis. The results showed that the expression of VEGF, SDF, SCF, MMP-1, MMP-2 and MMP-3 in the sh-H19 group decreased significantly compared with the sh-H19 NC group (Fig. 3h). The above results suggested that the modified preservative fluid preserved autologous blood through up-regulating H19 expression promoted angiogenesis and fibroblast migration, and increased the secretion of extracellular matrix, thereby significantly promoting wound healing in diabetic mice.

### H19 promotes histone methylation by recruiting EZH2

**Fig. 4:**
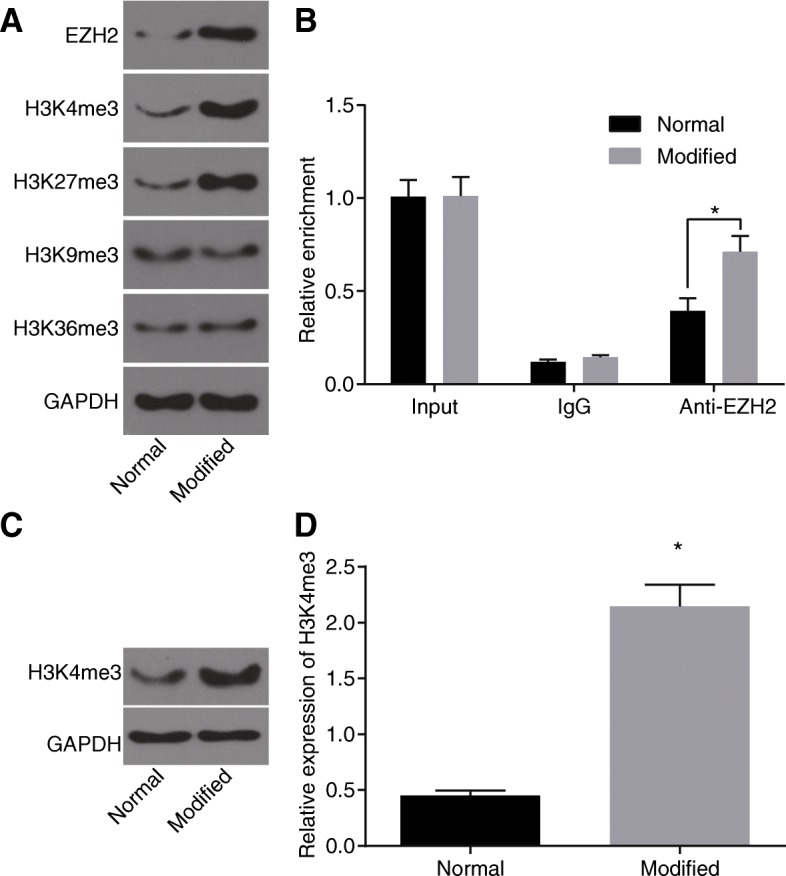
H19 promotes histone methylation by recruiting EZH2. **a** expression of relevant proteins binding to H19 in each group detected by western blot analysis after RNA-pull down assay; **b** expression of H19 combining with EZH2 in each group detected by RT-qPCR after RIP assay; **c** protein bands of H3K4me3 and GAPDH binding to EZH2 in each group determined by western blot analysis; **d** relative expression of H3K4me3 using western blot analysis; *, *p* < 0.05, compared with the normal group; The results were measurement data, which were presented as the mean ± standard deviation. Comparisons between two groups were analyzed by *t*-test. The experiment was repeated 3 times; normal, autologous blood preserved in standard preservative fluid; modified, autologous blood preserved in modified preservative fluid; RIP, RNA immunoprecipitation; RT-qPCR, reverse transcription quantitative polymerase chain reaction; EZH2, enhancer of zeste homolog 2; GAPDH, glyceraldehyde-3-phosphate dehydrogenase

It has been shown that H19 can interact with EZH2 [27], which has been demonstrated in the present study using RNA-pull down assay in diabetic fibroblasts. In addition, results of western blot analysis, which was used to determine the expression of relevant proteins binding to H19, suggested that in comparison with the normal group, the modified group exhibited elevated expression of EZH2, H3K4me3, and H3K27me3 but did not show significant changes in the expression of H3K9me3 and H3K36me3 (Fig. 4a). Compared with the standard preservative fluid, the modified preservative fluid preserved autologous blood could significantly recruit EZH2 and expression of H3K27me3 and H3K4me3 was upregulated. The interaction between H3K27me3 and diabetes has been extensively explored [28, 29]. Thus, H3K4me3 was selected as the study object in the current study. Results of RIP with EZH2 as the antibody showed that in contrast to the normal group, the expression of H19 binding to EZH2 was raised in the modified group (Fig. 4b). And then, COIP assay with EZH2 as the antibody was performed to further verify the relationship between EZH2 and H3K4me3. And western blot analysis was conducted to quantify the precipitated H3K4me3, finding that when compared with the normal group, the modified group presented increased expression of precipitated H3K4me3 (Fig. 4c, d). EZH2 was found to combine with H3K4 and lead to the trimethylation of H3K4 and autologous blood preserved in modified preservative fluid had upregulated expression of H3K4me3. The above results showed that the modified preservative fluid preserved autologous blood promoted histone H3K4 trimethylation by H19 recruiting EZH2.

### H19 epigenetically promotes the expression of HIF-1α

**Fig. 5:**
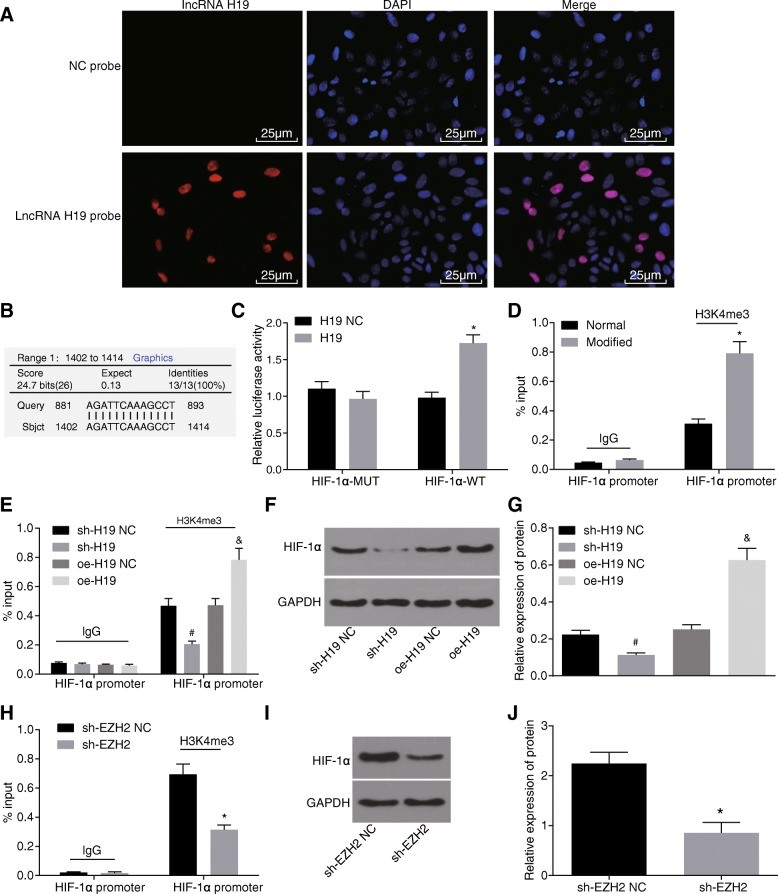
H19 could bind to HIF-1α gene promoter region and increase the expression of HIF-1α by triggering HIF-1α to recruit EZH2 and H3K4me3. **a** the location of H19 in fibroblasts detected by FISH; **b** the binding of H19 to HIF-1α gene promoter region predicted by bioinformatics website; **c** dual luciferase reporter gene assay further verified the targeting relationship of H19 to HIF-1α; **d-e** enrichment of H3K4me3 in HIF-1α gene promoter region by ChIP assay; **f-g** expression of HIF-1α after silencing and overexpressing H19; **h** enrichment of H3K4me3 in HIF-1α gene promoter region after EZH2 knockout by ChIP assay; **i-j** expression of HIF-1α in fibroblasts after EZH2 knockout by western blot analysis; *, *p* < 0.05, compared with the NC group or normal group; #, *p* < 0.05, compared with the sh-H19 NC group; &, *p* < 0.05, compared with the oe-H19 NC group. The above results were measurement data, which were presented as the mean ± standard deviation. Comparisons between two groups were analyzed by *t*-test. The experiment was repeated 3 times; ChIP, Chromatin Immunoprecipitation; GAPDH, glyceraldehyde-3-phosphate dehydrogenase; EZH2, enhancer of zeste homolog 2; NC, negative control; HIF-1α, hypoxia-inducible factor-1α; FISH, fluorescence in situ hybridization

During wound healing, local tissues were under anoxic condition, when the hypoxia inducible factor HIF-1α in the fibroblasts in the wound skin tissue was up-regulated. Results of FISH to determine the localization of H19 in fibroblasts revealed that H19 located in nucleus (Fig. 5a). Bioinformatics website (https://www.ncbi.nlm.nih.gov/) predicted the binding of H19 to HIF-1α gene promoter region. The results showed that H19 was complementary to HIF-1α gene promoter region (Fig. 5b). In order to verify the binding of H19 to HIF-1α gene promoter region, dual luciferase reporter gene assay was conducted. Compared with oe-H19 NC, the luciferase activity of HIF-1α-WT group increased significantly, and there was no significant change in HIF-1α-MUT group (Fig. 5c). The results showed that H19 could bind to HIF-1α gene promoter region and promote the expression of HIF-1α. At the same time, we also carried out the ChIP experiment. The results showed that compared with the standard preservative fluid, the modified preservative fluid preserved autologous blood presented elevated enrichment of H3K4me3 in HIF-1α gene promoter region (Fig. 5d). After silencing and overexpressing H19 in fibroblasts, we performed ChIP experiments. The results showed that compared with the sh-H19 NC group, the sh-H19 group showed significantly reduced enrichment of H3K4me3 in HIF-1α gene promoter region. When compared with the oe-H19 NC group, the enrichment of H3K4me3 in HIF-1α gene promoter region was enhanced in the oe-H19 group (Fig. 5e). Western blot analysis detected the expression of HIF-1α after silencing and overexpressing H19 in fibroblasts. The results showed that the expression of HIF-1α in the sh-H19 group was significantly lower than that in the sh-H19 NC group and the oe-H19 group had increased expression of HIF-1α than the oe-H19 NC group (Fig. 5f, g). Also, the results of ChIP revealed that, after knockout of EZH2 in fibroblasts, the enrichment of H3K4me3 in HIF-1α gene promoter region was decreased in the sh-EZH2 group when compared with the sh-EZH2 NC group (Fig. 5h). After the knockout of EZH2, the expression of HIF-1α was reduced in the sh-EZH2 group when compared with the sh-EZH2 NC group, which was determine by western blot analysis (Fig. 5i, j). These results showed that H19 could bind to HIF-1α gene promoter region and promote histone H3K4 trimethylation by recruiting EZH2 to HIF-1α gene promoter region, thus increasing the expression of HIF-1α.

### H19 augments wound healing in diabetic mice by activating HIF-1α signaling pathway

**Fig. 6:**
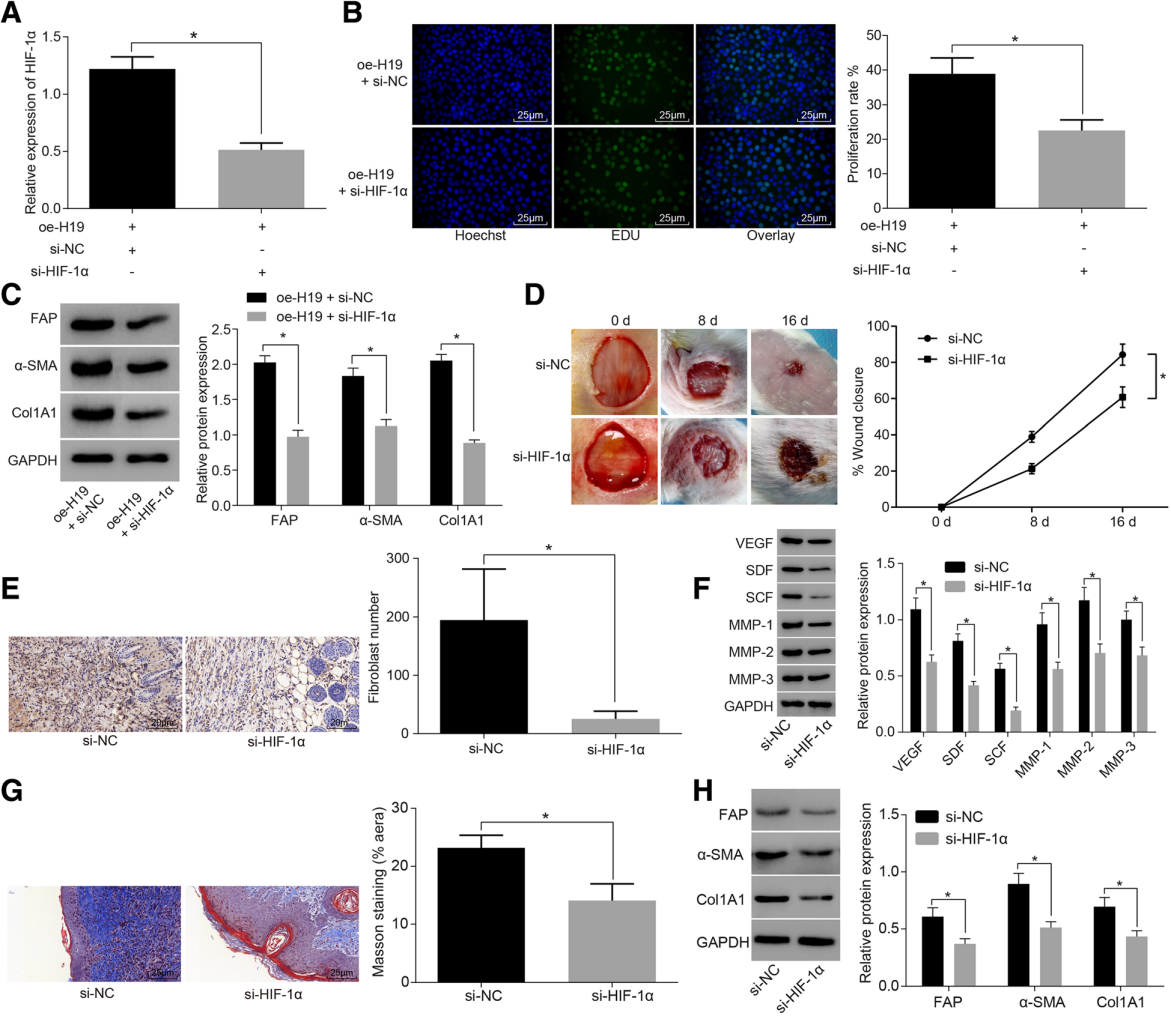
H19 facilitates wound healing in diabetic mice by activating HIF-1α signaling pathway. **a** RT-qPCR detection showing that expression of HIF-1α was significantly increased in response to H19 overexpression and si-HIF-1α co-transfection; **b** EDU staining (× 400) showing that fibroblast proliferation was inhibited in response to H19 overexpression and si-HIF-1α co-transfection; scale bar = 25 μm; **c** western blot analysis showing that the expression of activation related proteins (FAP, Col1A1 and α-SMA) in fibroblasts was significantly decreased in response to H19 overexpression and si-HIF-1α co-transfection; **d** wound healing speed was reduced in response to si-HIF-1α lentiviral injection; *N* = 8; **e** immunohistochemical staining (× 400) showing that number of fibroblasts was reduced in response to si-HIF-1α lentiviral injection; scale bar = 25 μm; N = 8; **f** western blot analysis showing that the expression of wound healing related proteins (VEGF, SDF, SCF, MMP-1, MMP-2 and MMP-3) was reduced; **g** Masson staining (× 400) showing that collagen content of skin tissue of diabetic mice was decreased; scale bar = 25 μm; N = 8; **h** western blot analysis showing that the expression of fibroblast activation related protein (FAP, Col1A1 and α-SMA) was reduced; *, *p* < 0.05, compared with the NC group; The above results were measurement data, which were presented as the mean ± standard deviation. Comparisons between two groups were analyzed by *t*-test. The speed of wound healing was analyzed by two-way analysis of variance. The experiment was repeated 3 times; RT-qPCR, reverse transcription quantitative polymerase chain reaction; HIF-1α, hypoxia-inducible factor-1α; DM, diabetes mellitus; EDU, ethynyl-2′-deoxyuridine; NC, negative control; VEGF, vascular endothelial growth factor; SDF, stromal cell-derived factor; SCF, stem cell factor; MMP, matrix metalloproteinase; α-SMA, α-smooth muscle actin

In order to detect H19 promoted wound healing in diabetic mice by activating HIF-1α signaling pathway, fibroblasts were co-transfected with H19, si-HIF-1α or si-NC. The expression of HIF-1α was detected by RT-qPCR. The results showed that the expression of HIF-1α in oe-H19 + si-HIF-1α group was significantly lower than that of oe-H19 + si-NC group (Fig. 6a). We also detected fibroblast proliferation by EDU test. The results showed that the proliferation ability of fibroblasts in the oe-H19 + si-HIF-1α was significantly lower than that of oe-H19 + si-NC group (Fig. 6b). Western blot analysis detected the expression of activation related protein in the fibroblasts after transfection. The results showed that compared with the e-H19 + si-NC group, the expression of FAP, Col1A1 and α-SMA was significantly decreased in the oe-H19 + si-HIF-1α (Fig. 6c). These results indicated that H19 promoted fibroblast activation by activating HIF-1α signaling pathway.

Further, we performed si-HIF-1α lentiviral injection on injured skin in diabetic mice, and performed modified preservative fluid preserved ABT, as well as detected the speed of wound healing. The results showed that compared with the si-NC group, the wound healing speed significantly reduced in the si-HIF-1α group (Fig. 6d). The immunohistochemical staining results of HSP (fibroblast markers) showed that the number of fibroblasts in the si-HIF-1α group was much lower than that in the si-NC group (Fig. 6e). The expression of wound healing related proteins was detected by Western blot analysis. The results showed that the expression of VEGF, SDF, SCF, MMP-1, MMP-2 and MMP-3 in the si-HIF-1α was significantly reduced compared with the si-NC group (Fig. 6f). At the same time, Masson staining was used to detect the collagen content of the skin tissue of diabetic mice. The results showed that the collagen content of skin tissue in the si-HIF-1α group decreased significantly compared with the si-NC group (Fig. 6g). Western blot analysis detected the expression of fibroblast activation related proteins. The results showed that the expression of FAP, Col1A1 and α-SMA in the si-HIF-1α group decreased significantly compared with the si-NC group (Fig. 6h). These results suggested that the modified preservative fluid preserved autologous blood could up-regulate H19 and activate the HIF-1α signaling pathway, whereby the activation of fibroblasts was enhanced and then wound healing in diabetic mice was augmented.

## Discussion

Wound healing is a dynamic and complicated process including inflammation, angiogenesis, coagulation, tissue formation and remodeling, which has been documented to be impaired by DM [30]. Recent evidence has shown that H19 is related to enhanced proliferation activity of keloid fibroblasts [11]. Fibroblasts, as a kind of mesenchymal cell, play a critical role in producing most extracellular matrix of connective tissues as well as wound healing [31]. A previous study has found that HIF-1α is implicated in skin wounds which have high level of hypoxia and HIF-1α is differentially expressed in skin wounds [32]. In this study, the results revealed that lncRNA H19 promoted fibroblast activation to improve wound healing in the diabetic mice by recruiting EZH2 mediated histone methylation and regulating HIF-1α signaling pathway.

One of our major findings was that the modified preservative fluid preserved ABT could promote the expression of H19 in fibroblasts, and better maintain oxygen-carrying capacity and oxygen release capacity as well as coagulation ability. A previous study revealed that lncRNA H19 served as a contributor to cardiac fibroblast proliferation as well as fibrosis by inhibiting DUSP5/ERK1/2 [33]. Besides, recent study reported that ABT exerted no negative influence on red blood cells for patients with DM receiving off-pump coronary artery bypass grafting [34]. Autologous blood point injection in patients with DM was found to significantly abate the symptoms of diabetic gastroparesis by means of reducing plasma motilin and gastrin levels [35]. Another study showed that haemoglobin A1c (HbA1c), a marker of DM reflecting the aging of RBC, was decreased in DM through ABT since the accumulated volume of donated blood could produce new RBCs in bone marrow [36]. As previously reported, blood transfusion also modified oxygen-carrying capacity as well as clinical signs of anaemia [37]. RBC transfusion is beneficial to increasing oxygen-carrying capacity of blood, improving tissue oxygenation, as well as hemostasis [38]. Moreover, research data showed that autologous blood could improve coagulation ability [39].

Additionally, our results found that H19 could bind to HIF-1α gene promoter region, promote HIF-1α histone H3K4me3 methylation by recruiting EZH2, and elevate the HIF-1α expression. A previous study found that hypoxia played a crucial role in increasing H19 expression in glioblastoma cells, which was associated with HIF-1α [40]. Another study indicated that HIF-1, as a transcriptional activator, is an important factor to regulate cell response to hypoxia as well as to enhance angiogenesis and improve skin wound healing [41]. It has been found that HIF-1α can bind to hypoxia response elements in the lncRNA-urothelial carcinoma associated 1 (UCA1) promoter [42]. Besides, it was known that histone methylation, one of the most widely and frequently studied post-transcriptional modifications, functions as a significant epigenetic event which has close relationship with cell fate determination and differentiation [43]. EZH2 is a methyltransferase and component of the polycomb repressive complex 2 and it is important for the epigenetic maintenance of the H3K27me3 repressive chromatin mark [44]. Recent evidence also showed that H19 could inhibit the promoter activity of BIK through the recruitment of EZH2 [45].

Furthermore, our study revealed that H19 promoted fibroblast activation by activating the HIF-1α signaling pathway in fibroblasts, thereby promoting wound healing in diabetic mice. The over-expression of HIF-1α notably increased the H19 expression in p53 hypoxic cells [46]. HIF-1 was reported to be decreased in diabetic wounds and served as a significant factor for promoting wound healing [47]. Besides, another study also indicated that HIF-1α exerted a positive effect on wound healing and decreased HIF-1α may result in impaired healing [19]. In addition, down-regulated H19 expression could inhibit fibroblast activation and lung fibrosis [48]. Myofibroblasts were found transient presence in wound healing and were widely known to be a crucial factor for wound closure as well as collagen synthesis [49].

## Conclusions

In conclusion, the key findings of the study showed that lncRNA H19 could recruit EZH2 mediated histone methylation and regulate HIF-1α signaling pathway to promote fibroblast activation, thereby improving wound healing of DM. Therefore, lncRNA H19 may potentially be a clinically viable target in the treatment of DM impaired wound healing. However, further studies are required to fully understand the specific mechanisms of H19 on wound healing impaired by DM through modified preservative fluid preserved ABT.

## Abbreviations

ABT: Autologous blood transfusion
APTT: Thromboplastin time
BLAST: Basic Local Alignment Search Tool
BSA: Bovine serum albumin
ChIP: Chromatin immunoprecipitation
DAB: Diaminobenzidine
D-D: D-dimer
DM: Diabetes mellitus
DMEM: Dulbecco’s modified Eagle’s medium
EdU: 5-ethynyl-2′-deoxyuridine
EZH: Enhancer of zeste
EZH2: Enhancer of zeste homolog 2
FIB: Fibrinogen
GAPDH: Glyceraldehyde-3-phosphate dehydrogenase
H19 NC: H19 negative control
HbA1c: Haemoglobin A1c
Hct: Hematocrit
HE: Hematoxylin and eosin
HGB: Hemoglobin
HIF-1α: Hypoxia-inducible factor-1α
lncRNA: Long non-coding RNA
MUT: Mutant
NC: Negative control
PAGE: Polyacrylamide gel electrophoresis
PBS: Phosphate buffered saline
PLT: Platelet
PT: Prothrombin time
PVDF: Polyvinylidene fluoride
RBC: Red blood cell
RIP: RNA Immunoprecipitation
RT-qPCR: Reverse transcription quantitative polymerase chain reaction
STZ: Streptozotocin
TT: Thrombin time
UCA1: Urothelial carcinoma associated 1
WT: Wild type

## References

[1] Tao SC, Rui BY, Wang QY, Zhou D, Zhang Y, Guo SC (2018). Extracellular vesicle-mimetic nanovesicles transport LncRNA-H19 as competing endogenous RNA for the treatment of diabetic wounds. Drug Deliv.

[2] Tsourdi E, Barthel A, Rietzsch H, Reichel A, Bornstein SR (2013). Current aspects in the pathophysiology and treatment of chronic wounds in diabetes mellitus. Biomed Res Int.

[3] Baltzis D, Eleftheriadou I, Veves A (2014). Pathogenesis and treatment of impaired wound healing in diabetes mellitus: new insights. Adv Ther.

[4] Xu J, Zgheib C, Hu J, Wu W, Zhang L, Liechty KW (2014). The role of microRNA-15b in the impaired angiogenesis in diabetic wounds. Wound Repair Regen.

[5] Okano T, Ohwada S, Sato Y, Sato N, Toyama Y, Nakasone Y, Ogawa T, Morishita Y (2001). Blood transfusions impair anastomotic wound healing, reduce luminol-dependent chemiluminescence, and increase interleukin-8. Hepato-Gastroenterology.

[6] Mazzucco L, Medici D, Serra M, Panizza R, Rivara G, Orecchia S, Libener R, Cattana E, Levis A, Betta PG (2004). The use of autologous platelet gel to treat difficult-to-heal wounds: a pilot study. Transfusion.

[7] Tao SC, Guo SC, Li M, Ke QF, Guo YP, Zhang CQ (2017). Chitosan wound dressings incorporating exosomes derived from MicroRNA-126-overexpressing synovium mesenchymal stem cells provide sustained release of exosomes and heal full-thickness skin defects in a diabetic rat model. Stem Cells Transl Med.

[8] Song X, Shan D, Chen J, Jing Q (2014). miRNAs and lncRNAs in vascular injury and remodeling. Sci China Life Sci.

[9] Liu Y, Liu DW (2016). Long non-coding RNA and wound healing. Zhonghua Shao Shang Za Zhi.

[10] Lu YF, Liu Y, Fu WM, Xu J, Wang B, Sun YX, Wu TY, Xu LL, Chan KM, Zhang JF (2017). Long noncoding RNA H19 accelerates tenogenic differentiation and promotes tendon healing through targeting miR-29b-3p and activating TGF-beta1 signaling. FASEB J.

[11] Zhang J, Liu CY, Wan Y, Peng L, Li WF, Qiu JX (2016). Long non-coding RNA H19 promotes the proliferation of fibroblasts in keloid scarring. Oncol Lett.

[12] Han YF, Sun TJ, Han YQ, Xu G, Liu J, Tao R (2015). Clinical perspectives on mesenchymal stem cells promoting wound healing in diabetes mellitus patients by inducing autophagy. Eur Rev Med Pharmacol Sci.

[13] de Almeida TF, de Castro Pires T, Monte-Alto-Costa A (2016). Blockade of glucocorticoid receptors improves cutaneous wound healing in stressed mice. Exp Biol Med (Maywood).

[14] Ruthenborg RJ, Ban JJ, Wazir A, Takeda N, Kim JW (2014). Regulation of wound healing and fibrosis by hypoxia and hypoxia-inducible factor-1. Mol Cells.

[15] Dong M, Fan XJ, Chen ZH, Wang TT, Li X, Chen J, Lin Q, Wen JY, Ma XK, Wei L (2014). Aberrant expression of enhancer of zeste homologue 2, correlated with HIF-1alpha, refines relapse risk and predicts poor outcome for breast cancer. Oncol Rep.

[16] Wang L, Jin Q, Lee JE, Su IH, Ge K (2010). Histone H3K27 methyltransferase Ezh2 represses Wnt genes to facilitate adipogenesis. Proc Natl Acad Sci U S A.

[17] Martinez-Zapata MJ, Marti-Carvajal AJ, Sola I, Exposito JA, Bolibar I, Rodriguez L, Garcia J, Zaror C. Autologous platelet-rich plasma for treating chronic wounds. Cochrane Database Syst Rev. 2016:CD006899. 10.1002/14651858.CD006899.pub3 PMC9308064 27223580

[18] Zhao YH, Ji TF, Luo Q, Yu JL (2017). Long non-coding RNA H19 induces hippocampal neuronal apoptosis via Wnt signaling in a streptozotocin-induced rat model of diabetes mellitus. Oncotarget.

[19] Mace KA, Yu DH, Paydar KZ, Boudreau N, Young DM (2007). Sustained expression of Hif-1alpha in the diabetic environment promotes angiogenesis and cutaneous wound repair. Wound Repair Regen.

[20] Marrotte EJ, Chen DD, Hakim JS, Chen AF (2010). Manganese superoxide dismutase expression in endothelial progenitor cells accelerates wound healing in diabetic mice. J Clin Invest.

[21] Quan C, Cho MK, Shao Y, Mianecki LE, Liao E, Perry D, Quan T (2015). Dermal fibroblast expression of stromal cell-derived factor-1 (SDF-1) promotes epidermal keratinocyte proliferation in normal and diseased skin. Protein Cell.

[22] Dunagin M, Cabili MN, Rinn J, Raj A (2015). Visualization of lncRNA by single-molecule fluorescence in situ hybridization. Methods Mol Biol.

[23] Pachnis V, Belayew A, Tilghman SM (1984). Locus unlinked to alpha-fetoprotein under the control of the murine raf and Rif genes. Proc Natl Acad Sci U S A.

[24] Jia P, Cai H, Liu X, Chen J, Ma J, Wang P, Liu Y, Zheng J, Xue Y (2016). Long non-coding RNA H19 regulates glioma angiogenesis and the biological behavior of glioma-associated endothelial cells by inhibiting microRNA-29a. Cancer Lett.

[25] Ding GL, Wang FF, Shu J, Tian S, Jiang Y, Zhang D, Wang N, Luo Q, Zhang Y, Jin F (2012). Transgenerational glucose intolerance with Igf2/H19 epigenetic alterations in mouse islet induced by intrauterine hyperglycemia. Diabetes.

[26] Su R, Wang C, Feng H, Lin L, Liu X, Wei Y, Yang H (2016). Alteration in expression and methylation of IGF2/H19 in placenta and umbilical cord blood are associated with macrosomia exposed to intrauterine hyperglycemia. PLoS One.

[27] Luo M, Li Z, Wang W, Zeng Y, Liu Z, Qiu J (2013). Long non-coding RNA H19 increases bladder cancer metastasis by associating with EZH2 and inhibiting E-cadherin expression. Cancer Lett.

[28] Manea SA, Antonescu ML, Fenyo IM, Raicu M, Simionescu M, Manea A (2018). Epigenetic regulation of vascular NADPH oxidase expression and reactive oxygen species production by histone deacetylase-dependent mechanisms in experimental diabetes. Redox Biol.

[29] Duraisamy AJ, Mishra M, Kowluru RA (2017). Crosstalk between histone and DNA methylation in regulation of retinal matrix Metalloproteinase-9 in diabetes. Invest Ophthalmol Vis Sci.

[30] Lim YC, Bhatt MP, Kwon MH, Park D, Na S, Kim YM, Ha KS (2015). Proinsulin C-peptide prevents impaired wound healing by activating angiogenesis in diabetes. J Invest Dermatol.

[31] Chiquet Matthias, Katsaros Christos, Kletsas Dimitris (2015). Multiple functions of gingival and mucoperiosteal fibroblasts in oral wound healing and repair. Periodontology 2000.

[32] Chen L, Gajendrareddy PK, DiPietro LA (2012). Differential expression of HIF-1alpha in skin and mucosal wounds. J Dent Res.

[33] Tao H, Cao W, Yang JJ, Shi KH, Zhou X, Liu LP, Li J (2016). Long noncoding RNA H19 controls DUSP5/ERK1/2 axis in cardiac fibroblast proliferation and fibrosis. Cardiovasc Pathol.

[34] Lin H, Zhang F, Pan Z, Chen X, Yu L, Yan M (2014). Effects of washed autologous blood transfusion on erythrocytic fragility in salvaged blood from diabetics. Zhonghua Yi Xue Za Zhi.

[35] Chen L, Zhang XF, Ku BQ, Wang XC, Ma C, Liang JY, Liu J (2012). Effects of acupoint injection of autologous blood on symptoms and plasma motilin and gastrin levels of diabetic gastroparesis patients. Zhen Ci Yan Jiu.

[36] Sugimoto T, Hashimoto M, Hayakawa I, Tokuno O, Ogino T, Okuno M, Hayashi N, Kawano S, Sugiyama D, Minami H (2014). Alterations in HbA1c resulting from the donation of autologous blood for elective surgery in patients with diabetes mellitus. Blood Transfus.

[37] Kisielewicz C, Self IA (2014). Canine and feline blood transfusions: controversies and recent advances in administration practices. Vet Anaesth Analg.

[38] Guzzetta NA (2011). Benefits and risks of red blood cell transfusion in pediatric patients undergoing cardiac surgery. Paediatr Anaesth.

[39] Takahashi N, Tsunematsu K, Sugawara H (2001). The use of the autologous blood transfusion to the respiratory surgery: autologous blood transfusion or no-blood transfusion surgery?. Kyobu Geka.

[40] Wu W, Hu Q, Nie E, Yu T, Wu Y, Zhi T, Jiang K, Shen F, Wang Y, Zhang J (2017). Hypoxia induces H19 expression through direct and indirect Hif-1alpha activity, promoting oncogenic effects in glioblastoma. Sci Rep.

[41] Du J, Liu L, Lay F, Wang Q, Dou C, Zhang X, Hosseini SM, Simon A, Rees DJ, Ahmed AK (2013). Combination of HIF-1alpha gene transfection and HIF-1-activated bone marrow-derived angiogenic cell infusion improves burn wound healing in aged mice. Gene Ther.

[42] Xue M, Li X, Li Z, Chen W (2014). Urothelial carcinoma associated 1 is a hypoxia-inducible factor-1alpha-targeted long noncoding RNA that enhances hypoxic bladder cancer cell proliferation, migration, and invasion. Tumour Biol.

[43] Zheng LW, Zhang BP, Xu RS, Xu X, Ye L, Zhou XD (2014). Bivalent histone modifications during tooth development. Int J Oral Sci.

[44] Yoo KH, Hennighausen L (2012). EZH2 methyltransferase and H3K27 methylation in breast cancer. Int J Biol Sci.

[45] Si X, Zang R, Zhang E, Liu Y, Shi X, Zhang E, Shao L, Li A, Yang N, Han X (2016). LncRNA H19 confers chemoresistance in ERalpha-positive breast cancer through epigenetic silencing of the pro-apoptotic gene BIK. Oncotarget.

[46] Matouk IJ, Mezan S, Mizrahi A, Ohana P, Abu-Lail R, Fellig Y, Degroot N, Galun E, Hochberg A (2010). The oncofetal H19 RNA connection: hypoxia, p53 and cancer. Biochim Biophys Acta.

[47] Sunkari VG, Lind F, Botusan IR, Kashif A, Liu ZJ, Yla-Herttuala S, Brismar K, Velazquez O, Catrina SB (2015). Hyperbaric oxygen therapy activates hypoxia-inducible factor 1 (HIF-1), which contributes to improved wound healing in diabetic mice. Wound Repair Regen.

[48] Lu Q, Guo Z, Xie W, Jin W, Zhu D, Chen S, Ren T. The lncRNA H19 mediates pulmonary fibrosis by regulating the miR-196a/COL1A1 Axis. Inflammation. 2018. 10.1007/s10753-018-0744-4 29411215

[49] Cheing GL, Li X, Huang L, Kwan RL, Cheung KK (2014). Pulsed electromagnetic fields (PEMF) promote early wound healing and myofibroblast proliferation in diabetic rats. Bioelectromagnetics.

